# Ginseng-derived nanoparticles inhibit lung cancer cell epithelial mesenchymal transition by repressing pentose phosphate pathway activity

**DOI:** 10.3389/fonc.2022.942020

**Published:** 2022-08-17

**Authors:** Lan Yang, Wen-qi Jin, Xiao-lei Tang, Shuai Zhang, Rui Ma, Da-qing Zhao, Li-wei Sun

**Affiliations:** ^1^ Research Center of Traditional Chinese Medicine, The Affiliated Hospital to Changchun University of Chinese Medicine, Changchun, China; ^2^ Jilin Ginseng Academy, Changchun University of Chinese Medicine, Changchun, China; ^3^ Key Laboratory of Active Substances and Biological Mechanisms of Ginseng Efficacy, Ministry of Education, Changchun, China

**Keywords:** ginseng-derived nanoparticles, EMT, lung cancer, pentose phosphate pathway, migration

## Abstract

It is unclear whether ginseng-derived nanoparticles (GDNPs) can prevent tumor cell epithelial-mesenchymal transition (EMT). Here, we describe typical characteristics of GDNPs and possible underlying mechanisms for GDNP antitumor activities. First, GDNPs particle sizes and morphology were determined using nanoparticle tracking analysis (NTA) and transmission electron microscopy (TEM), respectively, while cellular uptake of PKH67-labeled GDNPs was also assessed. Next, we evaluated GDNPs antitumor effects by determining whether GDNPs inhibited proliferation and migration of five tumor cell lines derived from different cell types. The results indicated that GDNPs most significantly inhibited proliferation and migration of lung cancer-derived tumor cells (A549, NCI-H1299). Moreover, GDNPs treatment also inhibited cell migration, invasion, clonal formation, and adhesion tube formation ability and reduced expression of EMT-related markers in A549 and NCI-H1299 cells in a dose-dependent manner. Meanwhile, Kaplan-Meier analysis of microarray data revealed that high-level thymidine phosphorylase (TP) production, which is associated with poor lung cancer prognosis, was inhibited by GDNPs treatment, as reflected by decreased secretion of overexpressed TP and downregulation of TP mRNA-level expression. In addition, proteomic analysis results indicated that GDNPs affected pentose phosphate pathway (PPP) activity, with ELISA results confirming that GDNPs significantly reduced levels of PPP metabolic intermediates. Results of this study also demonstrated that GDNPs-induced downregulation of TP expression led to PPP pathway inhibition and repression of lung cancer cell metastasis, warranting further studies of nano-drugs as a new and promising class of anti-cancer drugs.

## Introduction

Lung cancer, a major threat to human health and survival, accounts for the highest cancer incidence and mortality rates worldwide (1). In fact, nearly 80% of lung cancer patients are diagnosed during advanced stages of the disease, with very poor prognosis for most patients resulting from disease recurrence and distant metastasis. Non-small cell lung cancer (NSCLC) accounts for approximately 85% of all lung cancer cases (2, 3). During NSCLC disease progression a critical process occurs, epithelial mesenchymal transition (EMT), that endows the cells with enhanced migratory ability, invasiveness, and metastatic capability, thereby increasing cancer cell survival rates (4).

The root of *Panax ginseng* C.A. Meyer (Araliaceae) is famous for its many pharmacological properties that stem from regulatory activities of its constituent components of signal pathways related to inflammation, oxidative stress, angiogenesis, and cancer metastasis (5, 6). In recent years, it has been reported that ginseng extract has good efficacy as a cancer treatment, due to antitumor activities of components that include Rh2, Rg3, and Rg5 saponins. However, mechanisms underlying ginseng antitumor effects are still only partially understood (7–9).

Physiological cell-to-cell communication is essential for maintaining tissue homeostasis and biological integrity, with most cell-to-cell communication coordinated through multiple informational exchange mechanisms (10, 11). In recent years, nanovesicle-mediated cell communication has become a research hotspot, since nanovesicles can transfer diverse types of molecules from producing cells to target cells through extracellular transport as special extracellular vesicles (EVs) called exosomes (12). Plant exosome-like nanovesicles, nano-sized particles, are known to play a role in cell communication by transporting mRNA, miRNA, bioactive lipids, and multiple proteins between cells that allow different cells to work together as a functional unit. Importantly, nanovesicles can freely enter target cells and thus are viewed as a potentially useful vehicle for drug delivery (13–15). Recent studies have indicated that exosome-like nanoparticles released from edible plants (grapes, grapefruit, ginger, and carrots) have anti-inflammatory properties that appear to alleviate inflammatory bowel diseases (16–20), while ginseng-derived nanoparticles have been shown to exert an immunomodulatory effect on murine macrophages to inhibit tumor growth (21). Notably, lemon-derived nanovesicles have been shown to trigger TRAIL-mediated apoptosis that can effectively kill tumor cells (11).

Tumor cells can grow rapidly in an uncontrolled manner that is related to reprogramming of tumor cell metabolism. Unlike normal cells, which obtain energy from glycolysis and aerobic respiration, most tumor cells can also obtain energy through the pentose phosphate pathway (PPP), due to a phenomenon known as the Warburg effect. In fact, cancer cells are known to rely significantly on the PPP pathway to obtain energy and reducing equivalents needed for cell proliferation, invasiveness, drug resistance, and metastasis that enhance cell survival (22). Therefore, inhibition of the PPP pathway may significantly prevent tumor cell proliferation and metastasis (23). At the same time, cancer cells can also avoid metabolic regulatory roadblocks by deleting or acquiring key tumor suppressor genes and oncogenes, respectively (24, 25). For example, Twist1, a key transcription factor associated with tumor cell-associated EMT, promotes increased expression of thymidine phosphorylase (TP), a rate-limiting PPP enzyme belonging to the thymidine catabolic pathway. Importantly, studies have demonstrated that TP is overexpressed in human tumors, including those associated with non-small cell lung cancer (NSCLC), with TP expression level shown to be positively correlated with advanced-stage tumor metastasis and poor prognosis (26, 27).

In this study, antitumor effects of GDNPs on five different cancer cell lines were evaluated. The results demonstrated that GDNPs exhibited excellent antitumor effects against lung cancer-derived A549 and NCI-H1299 cells, warranting further development of nano-drugs for use as anti-cancer therapies.

## Materials and methods

### Cell culture

Cell lines used in this study, which included lines derived from hepatocellular carcinoma cells (HpG2), lung cancer cells (NCI-A549, hereafter referred to as A549 cells; NCI-H1299, hereafter referred to as H1299 cells), breast cancer cells (MCF-7), colorectal cancer cells (HCT-8), and pancreatic cancer cells (PANC-1), were obtained from KeyGen Biotech (Nanjing, China). All cells were cultured in RPMI 1640 or DMEM media supplemented with 10% fetal bovine serum under conditions of 37°C, 95% humidity, and 5% CO_2_ (hereafter referred to as standard cell culture conditions). No primary human tumor specimens were used in this study.

### Plasmid construction and cell transfection

The pcDNA3.1-TP plasmids were purchased from GeneCopoeia (Guangzhou, China). TP enzyme inhibitor (TPI) tipiracil (10μM, Meilunbio, China) was added to the medium for enzyme inhibition experiments. Plasmid vectors were transfected into cells using Lipofectamine 2000 (Invitrogen, Shanghai, China).

### Isolation of GDNPs

For isolation of GDNPs, fresh ginseng roots were purchased from Panax Ginseng Base Co. (Songyuan, Jilin, China). After roots were washed three times with deionized water at room temperature (RT), roots were pulverized using a juicer to yield juice. The resulting juice was centrifuged 300 × g for 30 min then was filtered through a 0.45-μm membrane and the supernatant was retained. Next, the supernatant was centrifuged at 2000 × *g* for 20 min and the resulting supernatant was retained and centrifuged at 10000 × *g* for 40 min through a 0.45-μm membrane to remove large particles and fibers. Thereafter, the supernatant was filtered through a 0.22-μm membrane then the resulting pellet that contained GDNPs was resuspended. The suspension was then concentrated by ultracentrifugation at 100000 × *g* for 60 min (Beckman at Optima XE-100, Beckman, USA). Next, the pellet was resuspended in 100 μL of pre-cooled vesicle isolation buffer (VIB, 20 mm MES, 2 mM CaCl_2_, 0.1 M NaCl, pH 6), then the protein concentration of the GDNPs preparation was determined using a BCA protein assay kit (12, 28).

### GDNPs characterization and nanoparticle tracking analysis

GDNPs size distribution and concentration were determined *via* nanoparticle tracking analysis (NTA), a method based on light scattering properties of particles. NTA was conducted using a Zetasizer Nano ZS90 system (Malvern Instruments, England) equipped with a blue laser (405 nm) according to the manufacturer’s instructions provided with the system.

### Transmission electron microscope analysis of GDNPs

For TEM, 10 μL of sample solution was loaded onto copper mesh-coated carbon film then the sample was allowed to adsorb to the film for 1 min at room temperature. After removal of the remaining solution by dabbing the film with filter paper, the adsorbed sample was negatively stained with 10 μL uranyl acetate for 1 min. Thereafter, the excess staining solution was gently removed by dabbing the edge of the copper mesh with filter paper then the mesh was allowed to air-dry. Imaging was performed using a TEM system (HT-7700, Hitachi, Japan) operated at 100 kV.

### PKH67 labeling of GDNPs

Isolated GDNPs were labeled with the green fluorescent, lipophilic dye PKH67 (Umibio, UR52303) according to the manufacturer’s protocol. Briefly, to prepare the fluorescent dye, 1 μL PKH67 and 9 μL Diluent C were mixed and added to GDNPs then the mixture was incubated in the dark at RT for 10 min. Next, 1 mL of PBS was added then the mixture was ultracentrifuged at 100000 × *g* for 17 min and the supernatant was discarded. The pellet containing fluorescently labeled GDNPs was washed three times with phosphate-buffered saline (PBS) and 10 μL was removed and analyzed for protein content using a BCA protein assay kit. Next, PKH67-labeled GDNPs were added to A549 cells (5 × 10^5^/well) grown to 70% confluence in wells of in 24-well sterile were incubated for 0 h, 6 h, or 12 h. After incubation, the co-cultured cells and GDNPs in wells of plates were washed three times with PBS, fixed in 4% paraformaldehyde (PFA) at RT for 30 min, washed three times with PBS, then were stained with DAPI for 5 min. Images were taken of cells viewed under a Nikon confocal microscope.

### Flow cytometry

An A549 cell suspension (2 × 10^6^ cells/well) was inoculated into 6-well plates followed by incubation of plates under normal culture conditions for 24 h. After incubation, PKH67-labeled GDNPs were added to cells followed by incubation as mentioned above for 6 or 12 h. Cells were collected then washed twice with PBS after centrifugation at 1000 rpm for 5 min. Cell samples were prepared and analyzed using a FACSAria™ II system (BD Biosciences, USA).

### Cell viability assay

After A549 and H1299 cells (4-6 × 10^3^/well) were cultured overnight in 96-well plates, various concentrations of GNDPs were added to wells then the plates were incubated for 48 h under normal cell culture conditions. Next, cytotoxicity was determined *via* MTT assay whereby 10 μL of MTT solution (5 mg/mL) was added to each well then plates were incubated for 4 h. Next, 100 μL DMSO solution was added per well then absorbance values of wells were measured at 490 nm using a spectrophotometer, with untreated cells serving as the negative control.

### Wound healing assay

Cells were seeded into wells of 24-well plates (5 × 10^5^ cells/well). to create a confluent monolayer. Next, a scratch wound was made in the monolayer in a straight line using a 200-μL pipette tip. The cells adhering to plates were removed and incubated with serum-free medium containing GDNPs (40 or 80 µg/mL). Images of wounds were captured under a microscope at 0 h and 48 h.

### Cell invasion assay

Cell invasion capacity was assessed using transwell plates containing membrane inserts with 0.8-μm pores (Corning, USA). Membrane inserts were coated with Matrigel (BD Biosciences) then complete culture medium (600 µL) was added to the lower chamber followed by insertion of membrane inserts into transwell plates. Next, 200 μL of cell suspension in serum-free medium (1 × 10^5^ cells/mL) with or without GDNPs was added to the upper chamber. After 24 h of incubation under standard cell culture conditions, non-invasive cells were removed from the upper chamber with a cotton swab then remaining cells on membrane inserts were fixed in 4% PFA for 15 min at RT. Next, cells were stained with 0.1% crystal violet solution for 20 min. Cell invasion was visually assessed microscopically based on counting of cells in five random fields per well.

### Cell adhesion assay

Cells adhesion was assessed using 96-well plates coated with Matrigel (30 μL/well). GDNPs-treated cell suspensions (5 × 10^4^) were added to wells then plates were incubated for 2 h. After non-adherent cells were removed by washing with PBS, remaining adherent cells were fixed in 4% PFA for 10 min then were stained with 0.1% crystal violet solution for 10 min then cell adhesion was observed under a microscope for five randomly selected fields per well.

### Colony formation assay

A549 and H1299 cell suspensions were seeded at 400 cells/well in 6-well plates then medium containing GDNPs was added to wells and replaced every 3 days. After 12 days of incubation, cell colonies that had formed were fixed in 4% PFA and stained with 0.1% crystal violet solution as described above. A digital camera was used to record images of stained cells.

### Tube formation assay

Wells of 96-well plates were coated with Matrigel (40 μL/well, 0.3 mg/ml, BD, USA) then plates were incubated under standard cell culture conditions for 2 h. Next, A549 and H1299 cell suspensions pretreated with GDNPs were added into Matrigel-coated plate wells then plates were incubated for 8 h under standard cell culture conditions. After incubation, tube formation ability of cells was evaluated based on images of cells obtained under a microscope.

### Immunofluorescent staining

Cells were seeded into wells of 24-well plates at an appropriate concentration and incubated at 37°C overnight. After treatment of cells with various concentrations of GDNPs for 24 h, cells were fixed in 4% PFA for 10 min, permeabilized in 0.1% Triton X-100 for 20 min, and washed three times with PBS-T (3 min/wash). Next, cells were blocked in 3% BSA for 1 h then were incubated overnight with primary antibody (anti-E-cadherin or anti-vimentin antibody) diluted 1:200 (Affinity) at 4°C. After three PBS washes, cells were incubated with Alexa Fluor 546-conjugated secondary antibody at RT for 2 h in the dark. Finally, nuclear staining was performed by incubation of cells with DAPI for 5 min. Images of labeled/stained cells were obtained using a laser scanning confocal microscope.

### Western blot analysis

Cells were lysed in radioimmunoprecipitation buffer (RIPA) containing halt protease and phosphatase inhibitor cocktail for 10 min on ice then were centrifuged at 12000 rpm for 15 min. Next, equal amounts of protein were separated by SDS-PAGE and transferred to PVDF membranes. Thereafter, membranes were blocked with 5% milk solution for 1 h then were incubated with primary antibodies specific for Twist1, E-cadherin, vimentin, or GAPDH (diluted 1:1000) at 4°C overnight. Next, membranes were washed then incubated with appropriate HRP-conjugated secondary antibodies at RT for 2 h. Finally, antibody-bound proteins were visualized on membranes using an enhanced chemiluminescent (ECL) kit.

### Quantitative real-time PCR

Total RNA isolation from A549 cells was performed using TRIzol Reagent (Invitrogen, USA) then cDNA synthesis was performed using a cDNA reverse transcription kit (TIANGEN, China). Next, quantitative real-time PCR (qRT-PCR) was performed using a 2× Taq Plus PCR MasterMix kit (TIANGEN, China) and PCR primers with sequences as listed in **Table 1**.

**Table 1:** PCR primer sequences.

Gene	Forward primer ((5′- to 3′))	Reverse primer (5′- to 3′)
**E-Cadherin**	CGAGAGCTACACGTTCACGG	GGGTGTCGAGGGAAAAATAGG
**Vimentin**	GACGCCATCAACACCGAGTT	CTTTGTCGTTGGTTAGCTGGT
**Twist1**	GTCCGCAGTCTTACGAGGAG	GCTTGAGGGTCTGAATCTTGCT
**snail**	TCGGAAGCCTAACTACAGCGA	AGATGAGCATTGGCAGCGAG
**Slug**	TGGTCAAGAAACATTTCAACG	GGTGAGGATCTCTGGTTTT GG
**TP**	CACATGCAGCAACCAGTCCA	GGGAGAAGCCTTGGTAGGGT
**GAPDH**	GGAGCGAGATCCCTCCAAAAT	GGCTGTTGTCATACTTCTCATGG

### Mass spectrometric analysis and bioinformatics analysis

Mass spectrometric assays were performed as previously reported with modifications (29). First, GDNPs frozen in liquid nitrogen were ground into powder then the powder was transferred to a centrifuge tube. Subsequently, a volume of lysate buffer equivalent to 4-times the powder volume was added to the powder then the mixture was sonicated three times on ice followed by centrifugation 2000 × *g* for 10 min to remove debris. Next, precooled (-20°C) 20% TCA was added to the supernatant followed by gentle mixing and incubation at -20°C for 2 h to allow precipitation to occur. Finally, the mixture was centrifuged at 12000 × *g* for 10 min then the supernatant was removed and the precipitate was washed three times with pre-cooled acetone (-20°C). The remaining protein-containing solid was dissolved in 8 M urea and the protein concentration was determined using a BCA kit according to the manufacturer’s instructions. Next, the Gene Ontology (GO)-annotated proteome was obtained using the UniProt-GOA database (https://www.ebi.ac.uk/GOA/). First, the identified protein ID was converted to a UniProt ID, then each protein was mapped to a GO ID based on its UniProt ID. Subsequently, each protein was assigned a Gene Ontology functional classification based on three categories: biological process (BP), cellular component (CC), or molecular function (MF). Kyoto Encyclopedia of Genes and Genomes (KEGG) biological pathway analysis (https://www.genome.jp/kegg/pathway.html).

### Enzyme-linked immunosorbent assay

Cell culture supernatants collected from A549 and H1299 cell cultures were analyzed for human thymidine phosphorylase (TP) and ribulose-5-phosphate (Ru5P) contents using ELISA kits (J&l Biological, China) in accordance with the manufacturer’s instructions.

### G6PD activity assay and NADP^+^/NADPH assay

A549 cells were seeded into wells containing culture medium and cultured for 24 h. Next, cells were lysed, cell lysates were centrifuged, then supernatants were collected. G6PD activity of supernatants was determined using a kit (Beyotime, China) according to the manufacturer’s instructions based on absorbance values measured at 450 nm. G6PD activity was proportional to NADPH concentration, as determined using a NADP^+^/NADPH Assay Kit (Beyotime, China) according to the manufacturer’s instructions.

### Cancer genome atlas -based data analysis

Representative immunohistochemical assay images were downloaded from the Human Protein Atlas (https://www.proteinatlas.org) image archive. Lung cancer patient data were downloaded from The Cancer Genome Atlas Lung Adenocarcinoma (TCGA-LUAD) database (https://portal.gdc.cancer.gov/projects/TCGA-LUAD) and additional data were obtained from The Tumor Immune Estimation Resource 2.0 (TIMER2.0) database, a publicly available repository of data related to tumor immunity and tumor gene expression (http://timer.cistrome.org/). For data analysis, first the Gene DE module was used to study differential expression of TP among various types of tumors then the Gene Corr module was used to verify correlations between TP and Twist1 expression based on Spearman analysis of LUAD data. Kaplan-Meier Plotter (https://kmplot.com/analysis/) was used for prognostic analysis. Based on the median TP expression level, patient samples were divided into two groups then the two groups were compared with respect to overall survival (OS), first-progression (FP) survival, and post-progression survival (PPS).

### Statistical analysis

For each study involving A549 and H1299 cells, three independent experiments were performed and statistical analysis of results was conducted using GraphPad Prism. All data in the study were evaluated using SPSS 24.0 statistical software. For each group were analyzed by one-way analysis. Measurement data were expressed as means ± SD (X ± SD). A difference of P< 0.05 was considered statistically significant.

## Results

### Isolation and characterization of ginseng-derived nanoparticles

**Figure 1:**
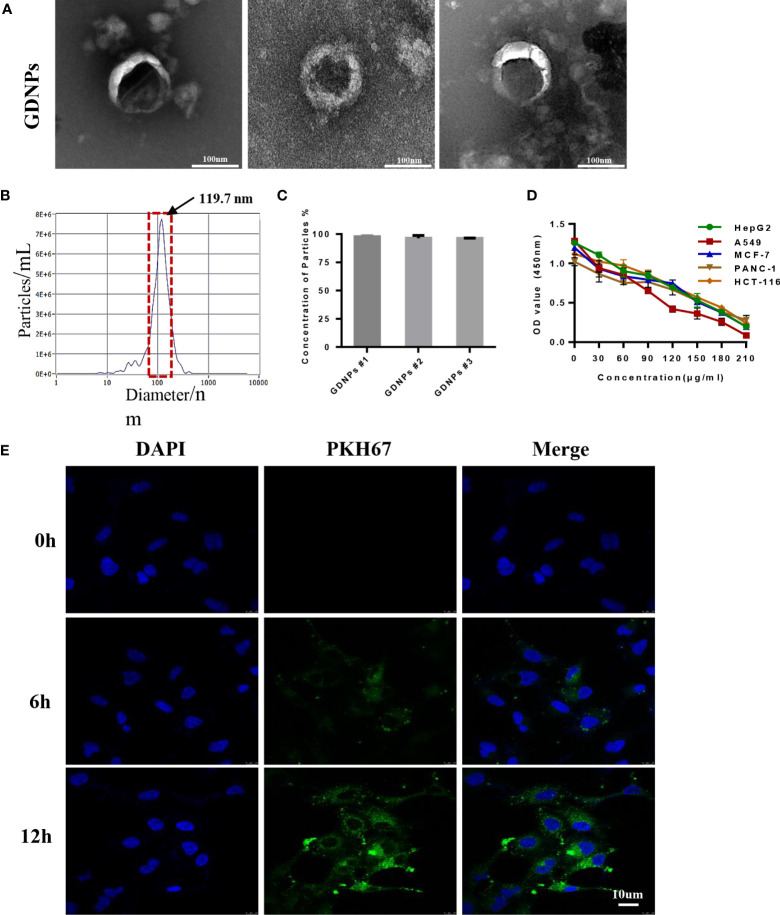
Characterization of nanoparticles derived from ginseng root. **(A)** The morphology of GDNPs was observed by TEM, scale bar = 100 nm (n = 3). **(B)** Nanoparticle tracking analysis (NTA)-based determination of GDNPs number and size distribution (n = 3). **(C)** GDNPs concentration expressed as percentage per batch (n = 3). **(D)** GDNPs effects on cell viability of HepG2, HCT-116, A549, PANC-1, and MCF-7 cells as analyzed *via* MTT assay. **(E)** Confocal microscopic image showing internalization of PKH-67-labeled GDPNs by A549 cells at indicated timepoints. Data are presented as the mean ± SD of three experiments.

First, we prepared plant-derived GDNPs by extracting juice from ginseng roots followed by processing of juice *via* centrifugation and filtration to isolate GDNPs. The result showed that GDNPs have an intact membrane and clearly visible the vesicle-like structure, and was generally spherical by Transmission electron microscopy (TEM) (**Figure 1A**). Meanwhile, Nanoparticle Tracking Analysis (NTA) revealed that the average diameter of GDNPs was about 119.7 nm, accounting for more than 93.5% of the total particle size, and also revealing a total particle concentration of 7.7E+6 particles/mL (**Supplementary Table 1**, **Figures 1B, C**). After GDNPs protein concentration was quantified using a BCA protein assay kit, GDNPS at various concentrations were evaluated for anticancer effects against five types of cancer cells (HepG2, HCT-116, A549, PANC-1, and MCF-7). Notably, results of MTT assays revealed that the GDNPs IC_50_ for A549 cells was approximately 80 µg/mL, a lower value than values obtained for the other cell lines (**Figure 1D**). To confirm this GDNPs effect on A549 cells, we also evaluated A549 cell uptake of PKH-67-labeled GDNPs by incubating A549 cells with 10 μg of PKH-67-labeled GDNPs for 0 h, 6 h, or 12 h. Immunofluorescence staining results demonstrated increased uptake with longer incubation times (**Figure 1E**, **Supplementary Figure 1A**). The results of flow cytometric analysis showed an increase in percentage of GDNPs-containing cells over time, as reflected by increasing absorption efficiency with time (**Supplementary Figures 1B, C**).

### GDNPs treatment inhibited cancer cell migration and proliferation activities

**Figure 2:**
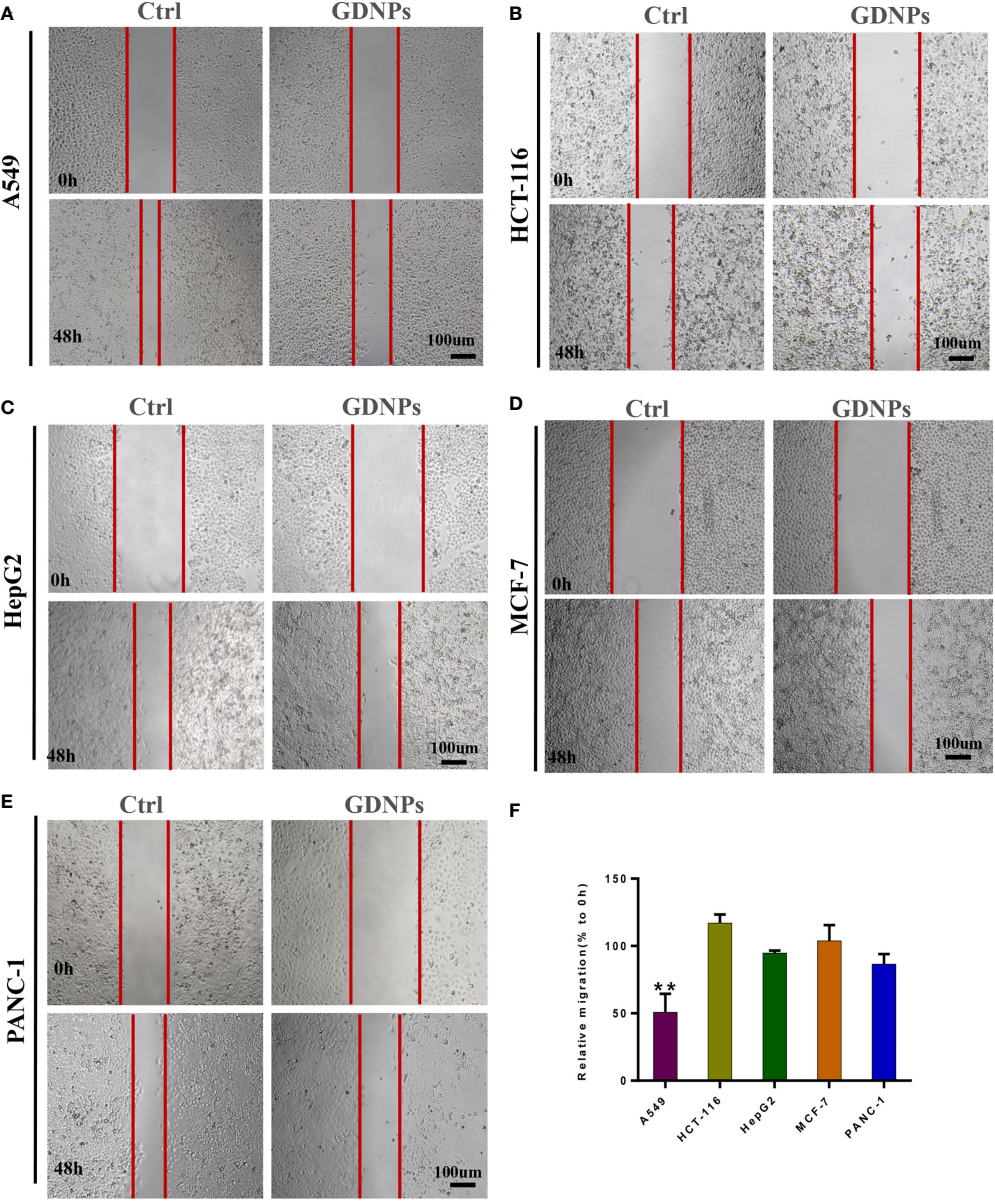
Effect of GDNPs on cancer cell migratory ability. **(A)** Analysis of A549 cells *via* wound-healing assay. **(B)** Analysis of HCT-116 cells *via* wound-healing assay. **(C)** Analysis of HepG2 cells *via* wound-healing assay. **(D)** Analysis of PANC-1 cells *via* wound-healing assay. **(E)** Analysis of MCF-7 cells *via* wound-healing assay. Images were acquired at 0 and 48 h. **(F)** Quantitative analysis of wound-healing assay data. Images were captured at 0 h and 48 h after wound formation, then the mean (%) change in width of the wound during healing was calculated by comparing wound width after 48-h healing (closure) to wound width at 0 h. Data are presented as the mean ± SD of three experiments (*P < 0.05).

Next, we studied GDNPs effects on cell migration using a wound-healing assay. Results of wound-healing assays indicated that GDNPs most significantly inhibited migration of A549 cells as compared to effects on migration of other cancer cell types (**Figures 2A**–**F**).

### GDNPs treatment inhibited migration, invasion, clonal formation, and adhesion of human NSCLC cells

**Figure 3:**
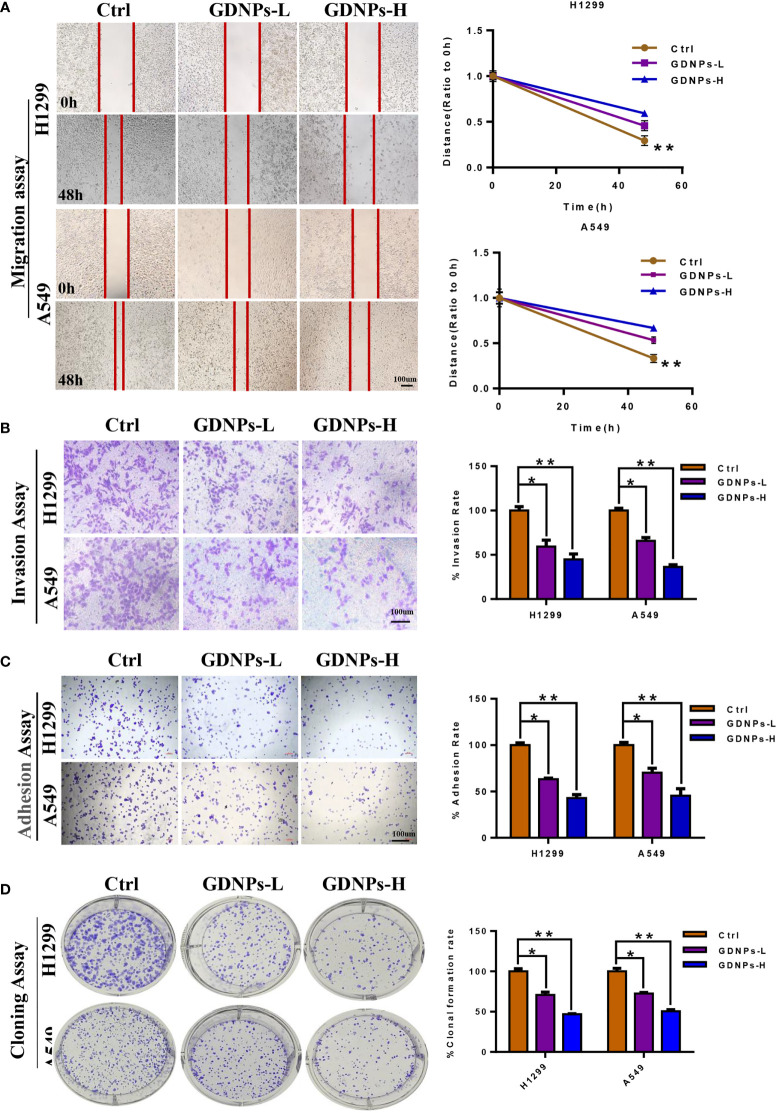
GDNPs inhibited human NSCLC cell migration, invasion, and colony formation. **(A)** GDNPs significantly inhibited migratory abilities of A549 and H1299 cells as compared with the untreated group cells as measured *via* the wound healing assay. **(B)** GDNPs significantly inhibited invasion abilities of two lung cancer cell lines as compared with the untreated group cells as measured vis the transwell assay. **(C)** GDNPs inhibited the adhesion of A549 and H1299 cells. **(D)** GDNPs inhibited colony formation by A549 and H1299 cells. GDNPs-H designates GDNPs at high concentration (60 μg/mL) and GDNPs-L designates GDNPs at low concentration (30 μg/mL). Statistical results are summarized in the right panel. Data are presented as the mean ± SD of three experiments (*P < 0.05 and **P < 0.01).

Based on findings of this study showing that GDNPs exerted excellent antitumor effects on A549 lung cancer cells, we next investigated whether GDNPs could exert additional antitumor effects on NSCLC cell-derived cells (A549 and H1299) in order to better understand mechanisms underlying GDNPs antitumor effects. First, GDNPs were assessed for their ability to inhibit migration and invasion activities of A549 and H1299 cells. Based on wound-healing assay results, GDNPs treatment resulted in significantly greater inhibition of migration of both cell types at 48 h as compared with results obtained for the untreated control group (**Figure 3A**). We next determined whether GDNPs could inhibit lung cancer cell invasion and adhesion activities. The results revealed that GDNPs were able to inhibit cell invasion and cell adherence (as based on cell numbers obtained in each assay) in a dose-dependent manner as compared to corresponding results obtained for the untreated group (**Figures 3B, C**). In addition, a cloning formation assay was performed that revealed that GDNPs inhibited cell clone formation ability in a dose-dependent manner (**Figure 3D**).

### GDNPs treatment of human NSCLC cells inhibited adhesion tube formation and reduced levels of EMT-related markers

**Figure 4:**
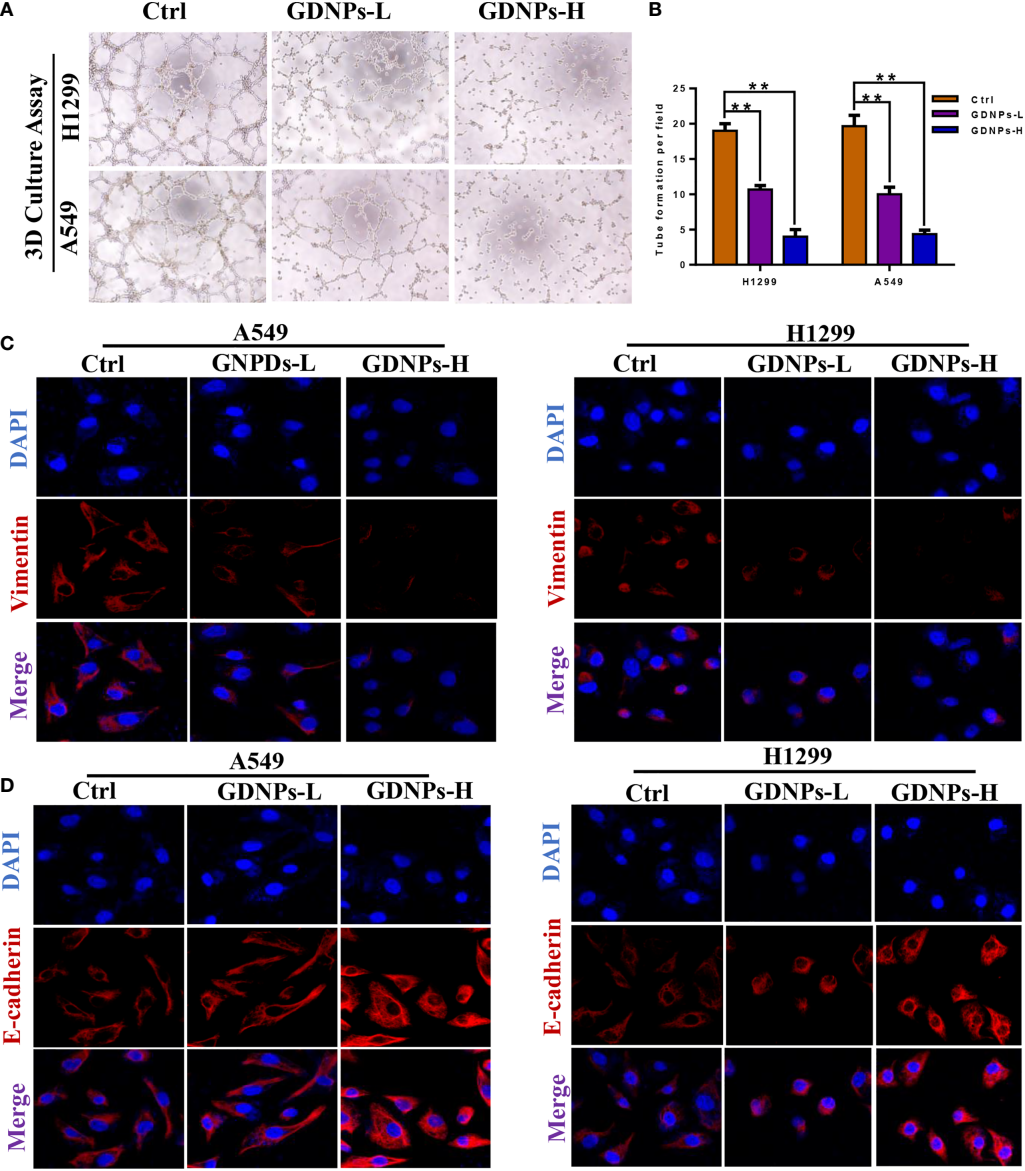
GDNPs treatment inhibited VM formation and reversed EMT biomarker changes. **(A, B)** Tube formation revealing decreased VM formation following GDNPs treatment, with statistical results summarized in panels to the right **(C, D)** Immunofluorescence assays revealed increased E-cadherin expression and decreased vimentin expression as compared with untreated group cells. Data are presented as the mean ± SD of three experiments (*P < 0.05).

**Figure 5:**
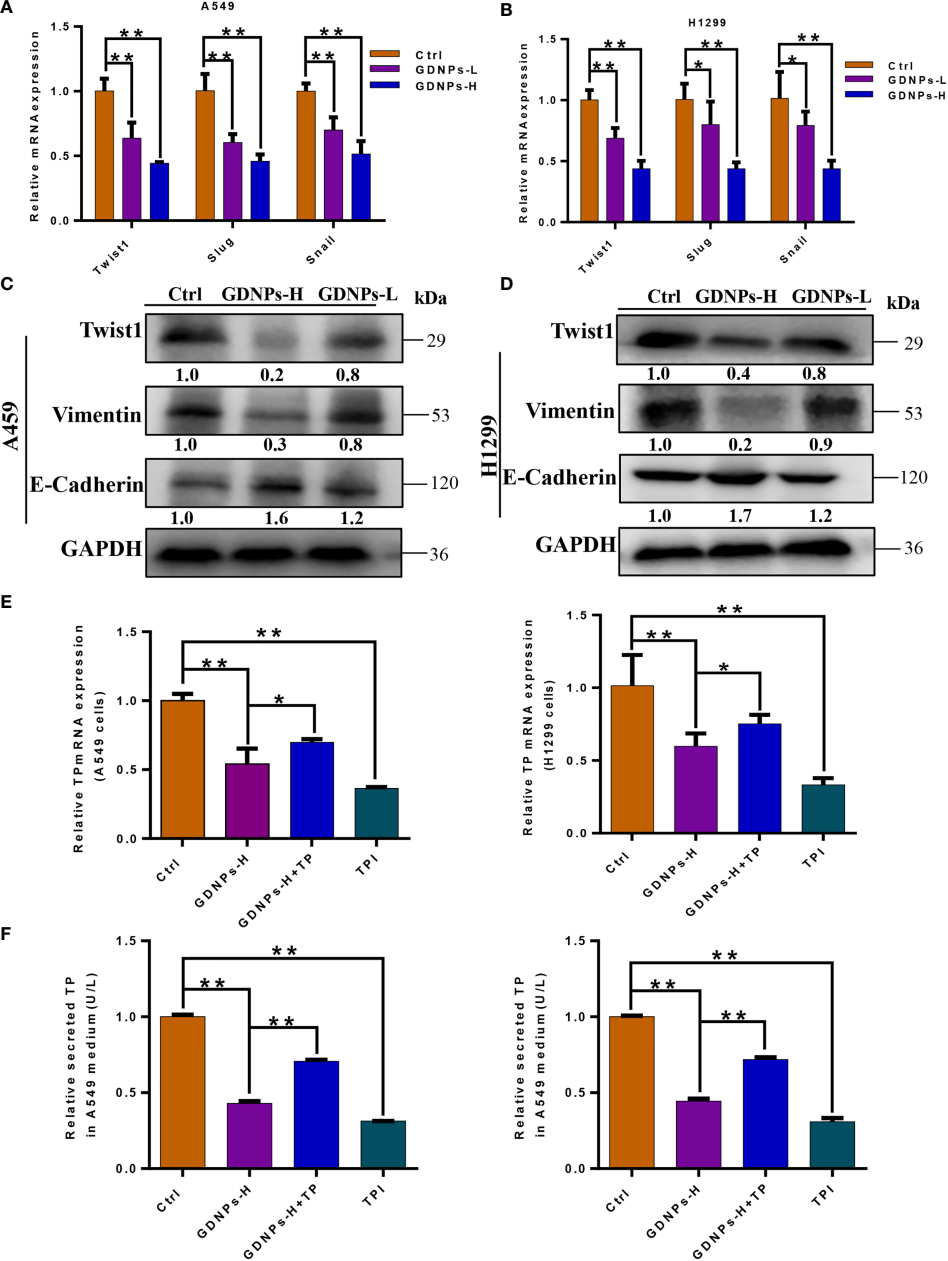
GDNPs treatment reduced levels of EMT-related makers and TP expression. **(A, B)** Expression of genes encoding epithelial and mesenchymal marker proteins in A549 and H1299 cells as measured *via* qRT-PCR after treatment of cells with GDNPs for 24 h. **(C, D)** Western blot results showing A549 and H1299 cells treated with GDNPs exhibited relatively decreased levels of vimentin and Twist1 and increased E-cadherin level as compared to untreated cells. **(E)** Results of qRT-PCR analysis showing expression levels of TP mRNA in A549 and H1299 cells. **(F)** Enzyme-linked immunosorbent assay results showing TP levels in medium of cultured cells. Data are presented as the mean ± SD of three experiments (*P < 0.05 and **P < 0.01).

Emerging evidence has confirmed that EMT is associated with increases in migration, invasion, and unique vasculogenic mimicry (VM) abilities associated with aggressive cancer cells such as NSCLC cells. To elucidate the mechanism associated with GDNPs-mediated inhibition of metastatic activities of A549 and H1299 cells, effects of GDNPs treatment on EMT were evaluated. First, A549 and H1299 cells were treated with various doses of exosomes for 24 h. Next, the GDNPs effect on cell VM formation was investigated using tube formation assay, with results revealing that GDNPs treatment significantly reduced the number of tube-like structures as compared to numbers in the untreated control group (**Figures 4A, B**). Additionally, immunofluorescence assay results showed that GDNPs treatment led to significantly increased expression of epithelial cell marker E-cadherin and decreased expression of mesenchymal cell marker vimentin in a dose-dependent manner (**Figures 4C, D**). Moreover, qRT-PCR results revealed GDNPs treatment-induced decreases in levels of transcription factor proteins Twist1, Snail, and Slug (**Figures 5A, B**), while Western blot results revealed that E-cadherin and vimentin protein expression levels were consistent with immunofluorescence assay results showing GDNPs inhibition of Twist1 expression (**Figures 5C, D**). Therefore, these results collectively suggest that GDNPs inhibited both EMT occurrence and VM channel formation in lung cancer cells.

### TP mRNA and protein levels were reduced after GDPNs treatment of A549 and H1299 cells

Previous studies had confirmed that main metabolic pathways associated with TP expression included the PPP and glycolysis, whereby TP expression was positively correlated with tumor stage, metastasis, and poor prognosis (30, 31). Due to the fact that TP enzymatic activity is required for angiogenesis, tumor growth and metastasis, we verified effects of GDNPs treatment on TP expression by measuring cell contents of TP protein and TP-encoding mRNA. ELISA results showed that GDNPs treatment of A549 and H1299 cells significantly inhibited cellular secretion of overexpressed TP protein, as did treatment of cells with TPI (**Figure 5E**); similar results were obtained for TP mRNA levels (**Figure 5F**). Therefore, we hypothesized that GDNPs inhibited lung cancer metastasis by downregulating TP as a potential anticancer strategy.

### Association of TP with clinical characteristics of lung cancer patients

**Figure 6:**
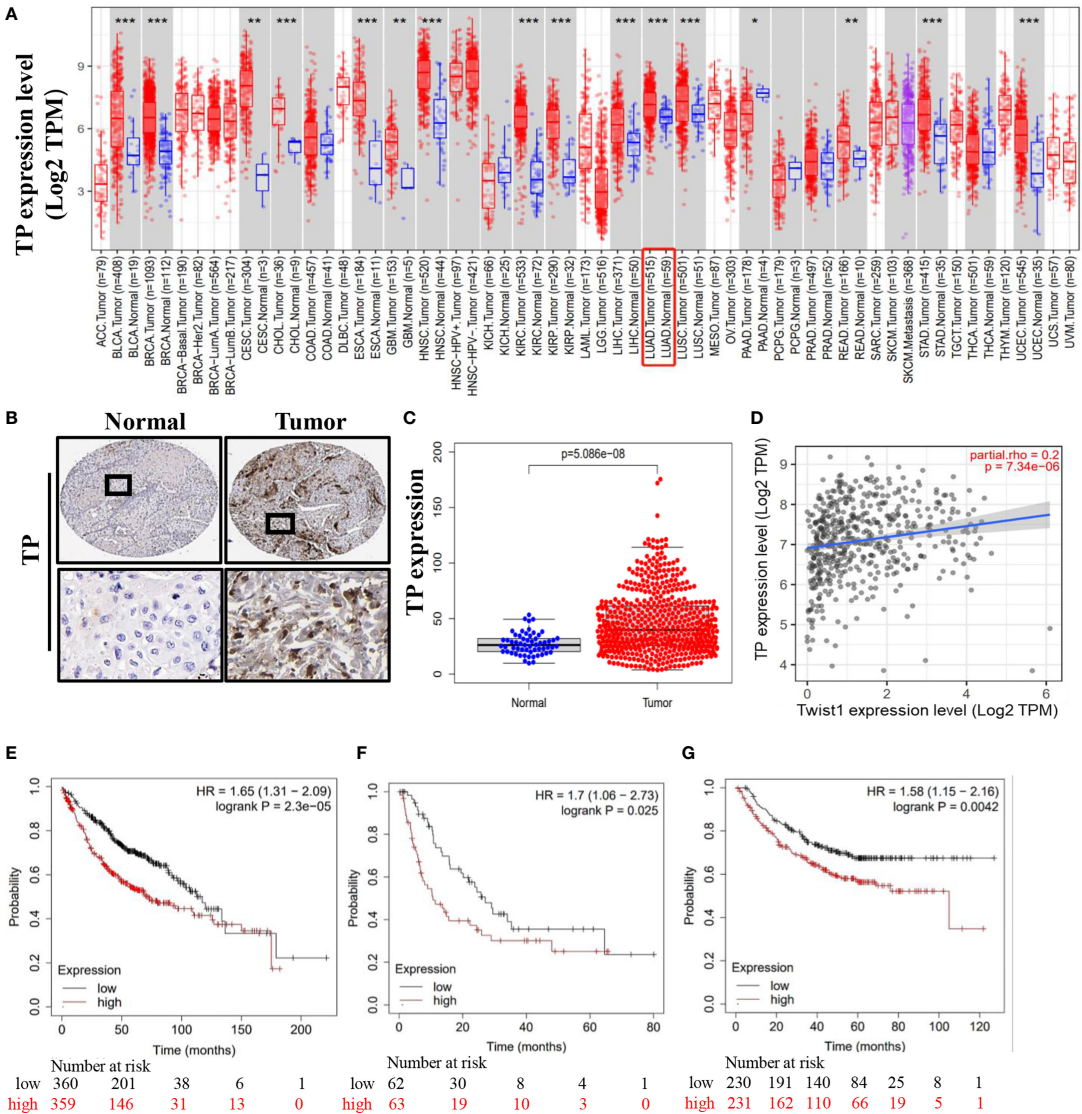
Lung cancer patient tissue TP levels as an indicator of poor prognosis. **(A)** High-level TP expression in lung cancer tissues. **(B, C)** Representative images derived from immunohistochemical staining of normal and lung cancer tumor tissues showing high tumor cell TP levels. **(D)** Correlation between TP and Twist1 expression levels. **(E)** Overall survival (OS). **(F)** First-progression survival (FP). **(G)** Post-progression survival (PPS). Data were obtained from the Human Protein Atlas and the TIMER2.0 database. (*P < 0.05 and **P < 0.01 and *** P < 0.001).

To further elucidate the role of TP in lung cancer patient survival, we compared TP expression levels of different tumor types to TP expression in normal tissues based on data obtained from the TIMER2.0 database. Results of this analysis demonstrated higher TP expression levels in lung cancer cells versus normal lung cells (**Figure 6A**). In addition, we also obtained data from the Human Protein Atlas database to compare TP expression levels, as determined using immunohistochemical analysis, between lung cancer tissues and adjacent normal tissues. The results revealed that TP was expressed at higher levels in tumor tissues than in adjacent normal (non-tumor) tissues (**Figure 6B**). Concurrently, analysis of TCGA results (Normal n = 59, Tumor n = 53) showed that TP mRNA-level expression was higher in tumor tissues than in normal tissues (**Figure 6C**). Next, we analyzed the relationship between TP expression and lung cancer patient prognosis. The results revealed that TP expression level was significantly and positively correlated with Twist1 expression in lung cancer cells (**Figure 6D**). Moreover, results of Kaplan–Meier analysis of microarray data showed that high TP expression was associated with poor prognosis, as reflected by low OS, PPS, and FP values associated with lung cancer patients (**Figures 6E-G**).

### Proteomic analysis of GDNPs

**Figure 7:**
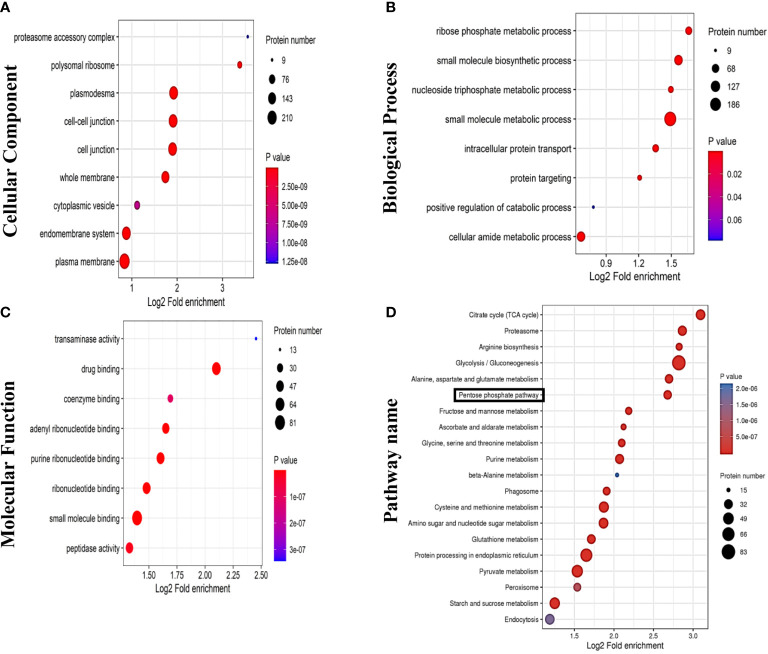
Results of proteomics analyses showing GDNPs effects on pentose phosphate pathway (PPP) activity. GO annotation results are listed under three main categories: **(A)** Biological Process. **(B)** Molecular Function. **(C)** Cellular Component. **(D)** KEGG pathway annotation analysis.

Analysis of GDNPs proteins led to identification of proteins belonging to all three GO categories of Biological Process (BP), Cellular Component (CC), and Molecular Function (MF). GO analysis results revealed that: BP protein functional terms were mainly associated positive regulation of catabolism, small molecule metabolism process, intracellular protein transport, ribose phosphate metabolic process, and small molecule biosynthesis process functions (**Figure 7A**); CC terms were mainly associated with cell-cell junction and endomembrane system functions (**Figure 7B**); and MF terms were mainly associated with small molecule binding, drug binding, coenzyme binding, and transferase activity functions (**Figure 7C**). We then investigated possible biological roles of exosome proteins based on KEGG pathway analysis. The results of KEGG analysis revealed that exosome proteins were mainly involved in several metabolic pathways, including the TCA cycle, PPP, pyruvate metabolism, and several other pathways (**Figure 7D**).

### GDNPs reduced PPP activity by blocking the warburg effect in NSCLC cells

**Figure 8:**
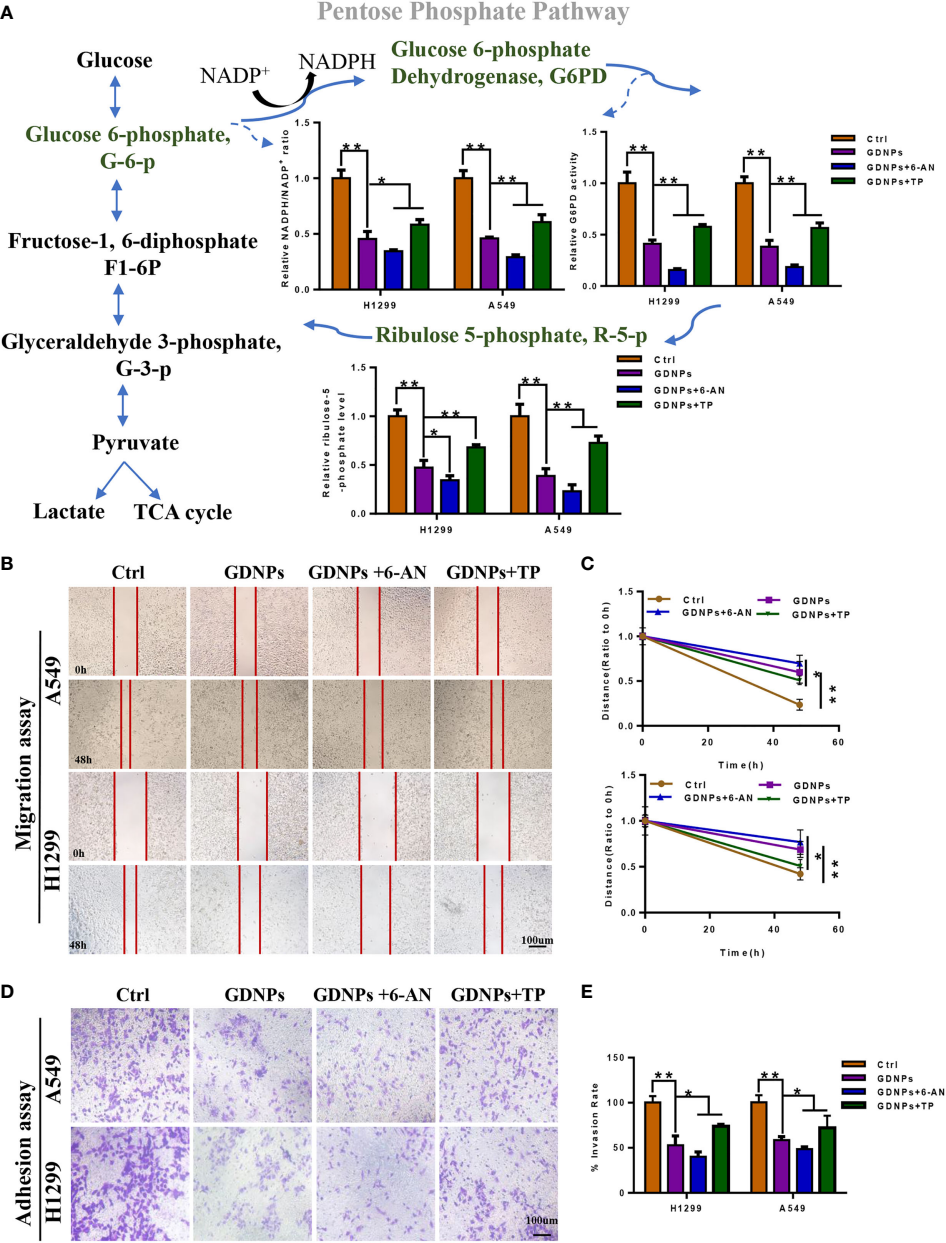
GDNPs and 6-AN can synergistically inhibit pentose phosphate pathway activities. **(A)** Contents of NADP**^+^**/NADPH and relative G6PD activities in A549 and H1299 cells of control and treated groups. Detection of contents of intermediate pentose phosphate pathway metabolites in A549 and H1299 cells treated with GDNPs and 6-AN. **(B-E)** Effect of GDNPs and 6-AN on migration and invasion of A549 and H1299 cells. Images were obtained at 200× magnification. Cells were then treated with 6-AN (50 µM). Data are presented as the mean ± SD of three experiments (*P < 0.05 and **P < 0.01).

G6PD is the rate-limiting enzyme within the PPP, a pathway that serves as the main source of reducing equivalents (NADPH) and pentose phosphate needed for tumor cell activities. To investigate effects of GDNPs on PPP metabolism in NSCLC cells, we added the effective antimetabolic drug aminonicotinamide (6-AN, G6PD inhibitor) and TP expression to investigate GDNP effects on cellular levels of NADPH and PPP rate-limiting enzyme G6PD and production of PPP intermediate metabolite R-5-P. The results showed that, as compared with the control, treatment of cells with GDNPs significantly inhibited G6PD expression and reduced cellular NADPH levels (as reflected by a decrease in the NADP+/NADPH ratio). Meanwhile, compared with the GDNPs treatment group, with more pronounced inhibition observed when cells were treated with both GDNPs and 6-AN than when either treatment alone, but TP expression group attenuated the inhibitory effect of GDNPs. In addition, we measured levels of the PPP intermediate metabolite R-5-P. The results showed that GDNPs could effectively inhibit production of R-5-P, with the combination of GDNPS and 6-AN also significantly reducing production of intermediate metabolites (**Figure 8A**). Furthermore, results showing the effect of PPP activity on migration of lung cancer cells revealed that treatment of A549 and H1299 cells with GDNPs in combination with 6-AN significantly inhibited cell migration and TP expression group attenuated the inhibitory effect of GDNPs (relative to results obtained for the GDNPs group) (**Figures 8B-E**). Taken together, these results suggest that PPP is one of the key metabolic pathways affected by GDNPs -induced downregulation of TP expression.

## Discussion

Lung cancer, a life-threatening oncological disease, is the leading cause of cancer-related deaths worldwide. It is well known that the majority of oncologic-related deaths are not caused by primary tumors but are instead caused by metastatic tumors (32, 33). The core process that is generally thought to drive tumor metastasis is the epithelial-mesenchymal transition (EMT), a process by which cells lose epithelial characteristics associated with low metastatic potential and gain mesenchymal cell characteristics associated with high metastatic potential (34). Main EMT hallmarks include loss of functional E-cadherin and overexpression of vimentin (35).

Extracellular vesicles (EVs) of mammalian cells, which can be internalized *via* endocytosis or phagocytosis, fuse with the target cell membrane then deliver their contents into the cytosol to influence cellular physiological and pathological processes (e.g., cancer, inflammatory bowel disease, and degenerative diseases) (36, 37). However, before EVs can be used to treat human diseases, challenges remain despite recent progress made in understanding EVs formation and secretion processes (38). Nevertheless, as compared to animal EVs, nanoparticles derived from edible plants are nontoxic, have low immunogenicity, can cross the blood-brain barrier, have target tissue specificity, can be internalized at high levels, and can be produced in large quantities (39, 40). To our knowledge, to date no applications of plant-derived nanoparticles for use in blocking cancer development have ever been described. This is surprising, due to the fact that from medicinal and functional perspectives, herbal nanoparticles appear to be easily internalized by mammalian cells and thus are amenable to experimental study for determining whether they can mediate cross-species communication (41).

In this study, we evaluated the integrity and size of GDNPs by TEM and NTA analysis. The results showed that GDNPs were in nanometer scale and have intact membranes with an average diameter of GDNPs was about 119.7 nm. Furthermore, our results revealed that GDNPs are stable and capable of functionally engaging in cross-species communication. In particular, here we demonstrated for the first time that GDNPs could be used to selectively inhibit growth of various types of cancer cells, while also significantly reducing migratory, infiltration, and clone formation abilities of A549 and H1299 lung cancer-derived cells. Moreover, GDNPs treatment also inhibited EMT and VM processes, upregulated E-cadherin expression, and decreased expression levels of vimentin and Twist1 to achieve an overall excellent antitumor effect.

Previous results reported by researchers associate with this study found that Twist1 production depends on TP-induced reprogramming of tumor metabolism to promote HCC cell VM formation and metastasis by invoking the pentose Warburg effect, thereby promoting tumor development (30). In this study, analysis of clinical data also revealed that high-level TP expression was positively correlated with low NSCLC patient prognostic indicator values for OS, PPS, and FP. The PPP, a major glucose metabolic pathway that is upregulated in cancer cells, depends on TP activity to convert pentose into glycerol-3-phosphate (G-3-P), a glycolytic pathway intermediate that links pentose metabolism with the glycolytic pathway. The results confirmed that GDNPs can inhibit PPP activity of lung cancer cells to reduce PPP-associated energy generation and PPP metabolic intermediates to ultimately inhibit tumor cell proliferation and other tumor-associated physiological processes. However, it remains to be determined whether ginseng-derived nanoparticles (GDNPs) inhibit lung cancer cell metastasis by downregulating TP expression through mechanisms involving miRNA- or protein-based PPP inhibition, warranting further study.

In summary, here we demonstrated for the first time that GDNPs could exert antitumor effects that effectively inhibited tumor cell proliferation, migration, invasion, and EMT occurrence. Mechanistically, antitumor GDNPs effects may be associated with downregulated TP expression resulting from PPP inhibition. These findings provide insights to guide future research on mechanisms underlying NSCLC progression and describe a new class of nano-drugs with potential activities for inhibiting cancer metastasis.

## Data availability statement

The original contributions presented in the study are included in the article/**Supplementary materials**. Further inquiries can be directed to the corresponding authors.

## Author contributions

LY, L-WS, and D-QZ conceived and designed the study. LY conducted the experiments and prepared the figures and tables. LY provided data analysis and wrote the original draft. W-QJ, X-LT, SZ, and RM supervised the research. All authors contributed to the article and approved the submitted version.

## Funding

This work was supported by the National Natural Science Foundation of China (Grant No. U20A20402, 82104428). The Science and Technology Development of Jilin Province (No. 20210304002YY).

## Conflict of interest

The authors declare that the research was conducted in the absence of any commercial or financial relationships that could be construed as a potential conflict of interest.

## Publisher’s note

All claims expressed in this article are solely those of the authors and do not necessarily represent those of their affiliated organizations, or those of the publisher, the editors and the reviewers. Any product that may be evaluated in this article, or claim that may be made by its manufacturer, is not guaranteed or endorsed by the publisher.
