# Supplementary figures and images for: Ginseng-derived nanoparticles inhibit lung cancer cell epithelial mesenchymal transition by repressing pentose phosphate pathway activity
Source: Front Oncol. 2022 Aug 17;12:942020. doi: 10.3389/fonc.2022.942020 (PMC9428604; doi:10.3389/fonc.2022.942020)

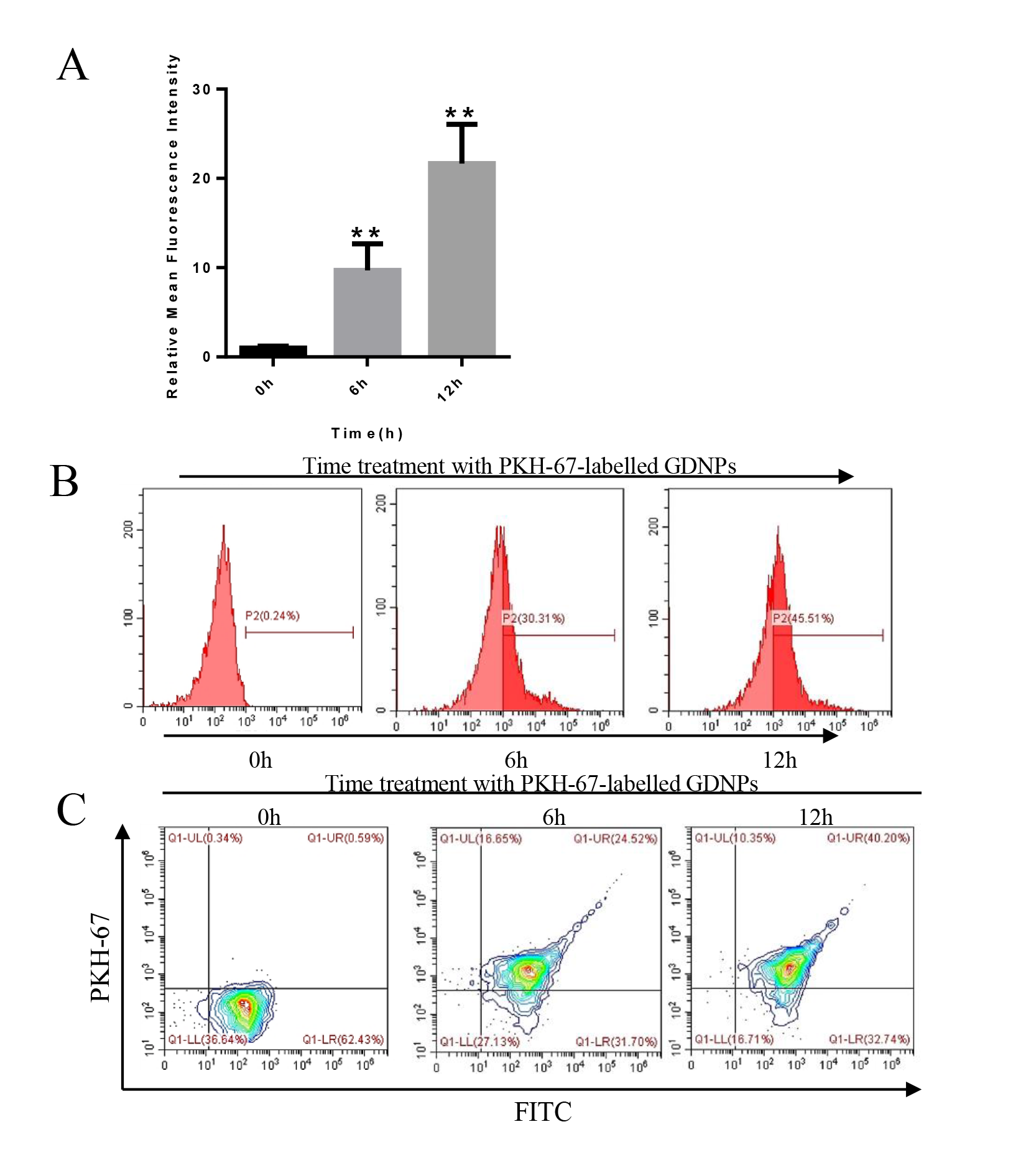

Supplement: Supplementary file 1 [file Image_1.tif]
