# Supplementary material for: Ginseng-derived nanoparticles inhibit lung cancer cell epithelial mesenchymal transition by repressing pentose phosphate pathway activity
Source: Front Oncol. 2022 Aug 17;12:942020. doi: 10.3389/fonc.2022.942020 (PMC9428604; doi:10.3389/fonc.2022.942020)
Supplement: Supplementary file 2 [file Table_1.docx]

| Diameter/nm | Particles/mL | FWHM/nm | Percentage |
| --- | --- | --- | --- |
| 119.7 | 7.7E+6 | 82.6 | 93.5 |
| 36.2 | 6.5E+5 | 16.4 | 5.8 |
| 7.1 | 5.0E+4 | 1.7 | 0.3 |
| 583.6 | 1.0E+4 | 119.0 | 0.0 |
| 777.8 | 8.8E+3 | 248.4 | 0.0 |

Supplementary Table 1. Results of NTA particle size peak analysis of GDNPs
